# Blood lipid profiles as a prognostic biomarker in idiopathic pulmonary fibrosis

**DOI:** 10.1186/s12931-024-02905-z

**Published:** 2024-07-18

**Authors:** Ju Hyun Oh, Ganghee Chae, Jin Woo Song

**Affiliations:** 1 Department of Pulmonology and Critical Care Medicine, Sanggye Paik Hospital, Inje University College of Medicine, Seoul, Republic of Korea; 2 Division of Pulmonology and Critical Care Medicine, Department of Internal Medicine, Ulsan University Hospital, University of Ulsan College of Medicine, Ulsan, Republic of Korea; 3 Division of Pulmonology and Critical Care Medicine, Department of Internal Medicine, Asan Medical Center, University of Ulsan College of Medicine, 88 Olympic-ro 43-gil, Songpagu, Seoul, 05505 Republic of Korea

**Keywords:** Apolipoprotein A-I, Idiopathic pulmonary fibrosis, GAP score, Mortality

## Abstract

**Background:**

Dysregulation of lipid metabolism is implicated in the pathogenesis of idiopathic pulmonary fibrosis (IPF). However, the association between the blood lipid profiles and the prognosis of IPF is not well defined. We aimed to identify the impacts of lipid profiles on prognosis in patients with IPF.

**Methods:**

Clinical data of 371 patients with IPF (145 and 226 in the derivation and validation cohorts, respectively), including serum lipid profiles (total cholesterol, triglyceride, high-density lipoprotein cholesterol, low-density lipoprotein cholesterol, apolipoprotein A-I [Apo A-I], and apolipoprotein B), were retrospectively collected. The association with mortality was analyzed using the Cox proportional hazard model.

**Results:**

In the derivation cohort, the mean age was 67.5 years, 86.2% were men, and 30.3% died during the follow-up (median: 18.0 months). Non-survivors showed lower lung function and greater gender-age-physiology scores than survivors. Among the serum lipid profiles, the levels of triglyceride and Apo A-I were significantly lower in non-survivors than in survivors. In the multivariate Cox analysis, low Apo A-I levels (< 140 mg/dL) were independently associated with the risk of mortality (hazard ratio 3.910, 95% confidence interval 1.170-13.069; *P* = 0.027), when adjusted for smoking history, body mass index, GAP score, and antifibrotic agent use. In both derivation and validation cohorts, patients with low Apo A-I levels (< 140 mg/dL) had worse survival (median survival: [derivation] 34.0 months vs. not reached, *P* = 0.003; [validation] 40.0 vs. 53.0 months, *P* = 0.027) than those with high Apo A-I levels in the Kaplan–Meier survival analysis.

**Conclusions:**

Our results indicate that low serum Apo A-1 levels are an independent predictor of mortality in patients with IPF, suggesting the utility of serum Apo A-I as a prognostic biomarker in IPF.

**Supplementary Information:**

The online version contains supplementary material available at 10.1186/s12931-024-02905-z.

## Background

Idiopathic pulmonary fibrosis (IPF) is a progressive fibrosing interstitial lung disease (ILD), characterized by poor prognosis with a median survival of approximately 3 years [1, 2]. Although the pathogenesis of IPF was not well defined, repeated alveolar epithelial injury leads to fibroblast proliferation and activation, followed by exaggerated deposition of extracellular matrix and the remodeling of the lung tissues in IPF [3]. Recent studies have also highlighted the role of epithelial-mesenchymal transition and the lung microbiome in the pathogenesis of IPF ​​ [4–6]. In addition, dysregulation of lipid metabolism is implicated also in the pathogenesis of IPF [7, 8]. Excessive accumulation of lipid droplets including free or esterified cholesterol in the lung epithelial cells induces inflammation and fibrosis of lung by promoting expression of collagens and release of cytokines such as transforming growth factor-β1 (TGF-β1), tumor necrosis factor-α (TNF-α), interleukin (IL)-1, IL-6, and matrix metalloproteinase (MMP)-3 [9].

The importance of lipid homeostasis has emerged in the development and progression of lung fibrosis [10–14]. Particularly, apolipoprotein A-I (Apo A-I), which plays a significant role in maintaining lipid homeostasis, was identified to have the ability to attenuate the induction of lung inflammation and fibrosis in previous studies [10, 15–17]. In the transgenic mice model, overexpression of Apo A-I significantly reduced the levels of TGF-β1, IL-1β, and TNF-α in lung tissues, and decreased silica-induced lung inflammation and fibrosis compared with control mice [16]. In the Multi-Ethnic Study of Atherosclerosis (MESA) study including 6,814 adults without cardiovascular disease, higher levels of serum high-density lipoprotein (HDL)-C and Apo A-I were associated with lesser lung high attenuation area (HAA) on cardiac computed tomography (CT) images, indicating subclinical inflammation and extracellular matrix remodeling in lung tissues [10]. Additionally, in a study including 28 patients with IPF, Kim et al. showed that Apo A-I levels in bronchoalveolar lavage fluid (BALF) significantly correlated with radiologic fibrosis score (r^2^= -0.23, *P* = 0.031) [15]. However, while these studies have revealed the protective role of Apo A-I against pulmonary fibrosis, most previous studies have been performed in mice models or only included a small number of patients with IPF [10, 15–17]. Furthermore, the association between lipid profiles including apolipoproteins and disease severity or prognosis in patients with IPF has not yet been clarified. Therefore, to elucidate the association of lipid profile with prognosis in patients with IPF, our study aimed to investigate the prognostic values of serum lipid profiles including Apo A-I levels in a large cohort of IPF patients.

## Materials and methods

### Study population

**Fig. 1:**
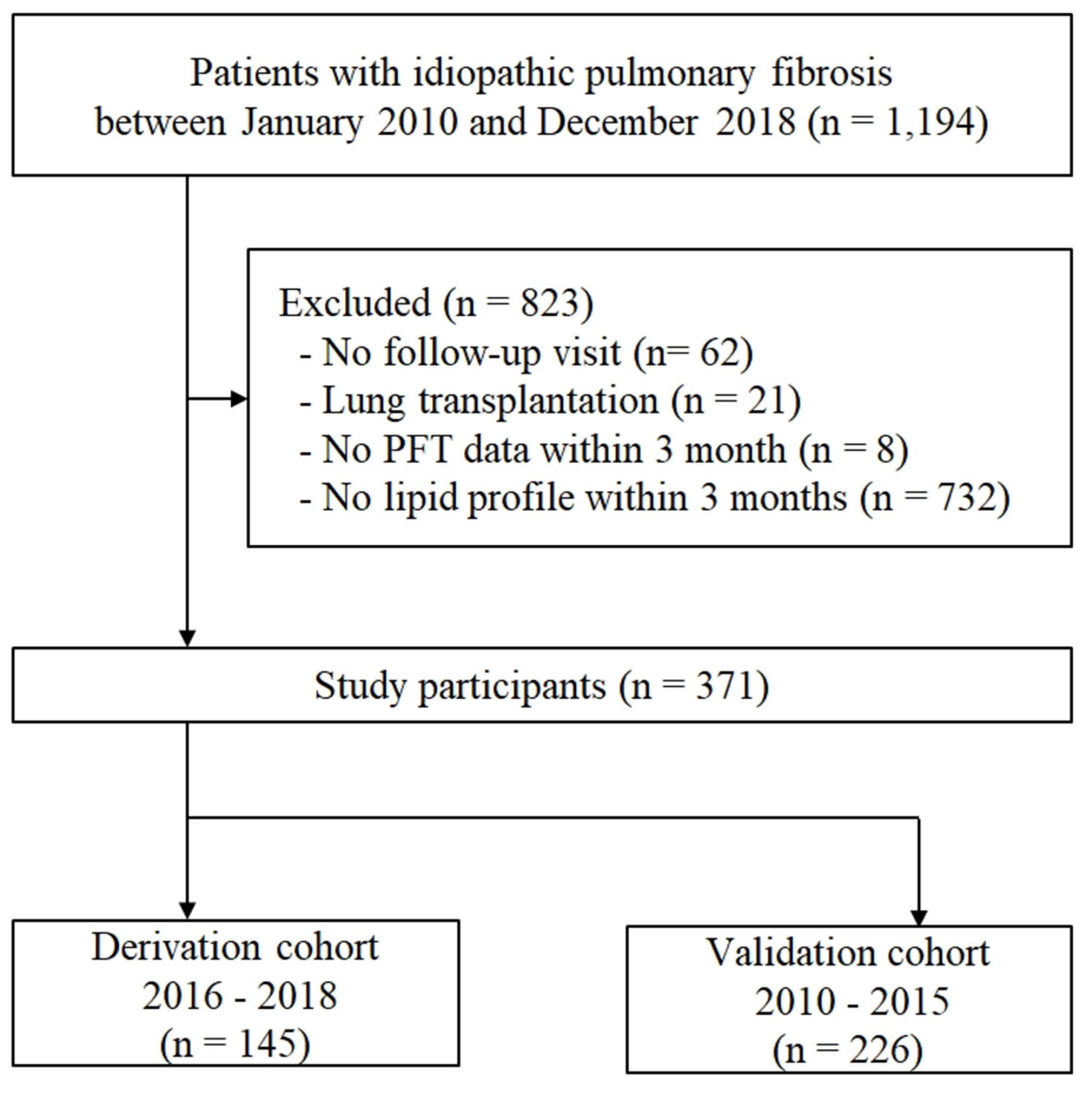
Flowchart of the study

A total of 1,194 patients with IPF diagnosed between January 2010 and December 2018 at Asan Medical Center, Seoul, Republic of Korea, were screened for this study. Patients were excluded from the study if they met any of the following criteria: lack of a follow-up visit after diagnosis (*n* = 62), underwent lung transplantation (*n* = 21) or absence of baseline lung function (*n* = 8) or insufficient laboratory data of lipid profiles, including apolipoproteins levels (*n* = 732) within 3 months from the date of IPF diagnosis. Finally, 371 patients with IPF were included in this study (biopsy-proven cases = 124). All patients were divided into the derivation (*n* = 145; between January 2016 and December 2018) and validation (*n* = 226; between January 2010 and December 2015) cohorts according to the time of IPF diagnosis (Fig. 1). All patients met the IPF diagnostic criteria of the 2018 American Thoracic Society (ATS), European Respiratory Society (ERS), Japanese Respiratory Society, and Latin American Thoracic Society statement [1]. This study was approved by the Institutional Review Board of Asan Medical Center (No.: 2021 − 0463), and the requirement for informed consent was waived due to the retrospective nature of the study.

### Clinical data

Clinical data were retrospectively collected by reviewing medical records and utilizing records from the National Health Insurance of Korea. All clinical parameters, including blood lipid profiles, were obtained within 3 months from the time of IPF diagnosis. Moreover, to assess lung function, spirometry [18] and diffusing capacity of the lung for carbon monoxide (DLco) [19] were measured according to the ATS/ERS recommendations, and expressed as percentages of the predicted values. The gender-age-physiology (GAP) score was calculated using clinical variables, which included gender, age, forced vital capacity (FVC), and DLco. The gender-age-physiology (GAP) score was calculated according to the approach proposed by Ley et al. [20]; using clinical variables including gender, age, forced vital capacity (FVC), and DLco, a total score was calculated (ranges: 0–8 points) and classified into stage I (0–3 points), stage II (4–5 points), or stage III (6–8 points).

### Measurement of lipid profile

Serum lipid profiles including total cholesterol, HDL, low-density lipoprotein (LDL), and triglyceride (TG), as well as levels of Apo A-I, and apolipoprotein B (Apo B) levels were obtained from patients after a minimum 8-hour fasting period. Total cholesterol, HDL, LDL, and TG levels were measured using an enzymatic colorimetric method using the Toshiba 200FR Neo analyzer (Toshiba Medical System Co., Ltd., Tokyo, Japan). Apo A-I and Apo B levels were determined using the turbidometric method using the Cobas Integra C-6000 analyzer (Roche Diagnostics, Basel, Switzerland).

### Statistical analysis

All continuous variables are represented as means ± standard deviations and categorical variables as numbers (percentages). To compare continuous variables, the Student’s t-test was used; however, the Chi-square test was used for categorical variables. The correlation between lipid profiles and baseline lung function or GAP score was assessed using Pearson’s correlation analysis. Furthermore, the follow-up period was calculated from the date of IPF diagnosis to the date of death or censoring (loss to follow-up or 30 June 2019). Cox proportional hazards analysis was also performed to identify risk factors for mortality among patients with IPF. Variables with a *P-*value of < 0.1 in the unadjusted Cox analysis were included in the multivariate Cox analysis, which employed a backward elimination, for the derivation cohort. The continuous lipid profile variables were dichotomized based on the cutoff values recommended by National Cholesterol Education Program Adult Treatment Panel III (NCEP ATP III) guidelines, defining optimal treatment ranges for TC (< 200 mg/dL), TG (< 150 mg/dL), HDL (≥ 40 mg/dL), and LDL (< 100 mg/dL) [21]. Additionally, the MaxStat package (Maximally selected Rank Statistics, version 0.7–25, Torsten Hothorn, 2017) was used to determine cutoff values for Apo A-I (≥ 140 mg/dL) and Apo B (≥ 80 mg/dL). The Kaplan–Meier survival analysis and log-rank test were used to compare survival outcomes, with a *P-*value < 0.05 considered statistically significant. All statistical analyses were performed using SPSS software (version 21.0; IBM Corporation, Somers, NY, USA), MedCalc Statistical Software (version 12.7.5; MedCalc Software bvba, Ostend, Belgium), and R statistical software (version 4.1.3, R Core Team, 2022).

## Results

### Baseline characteristics

**Fig. 2:**
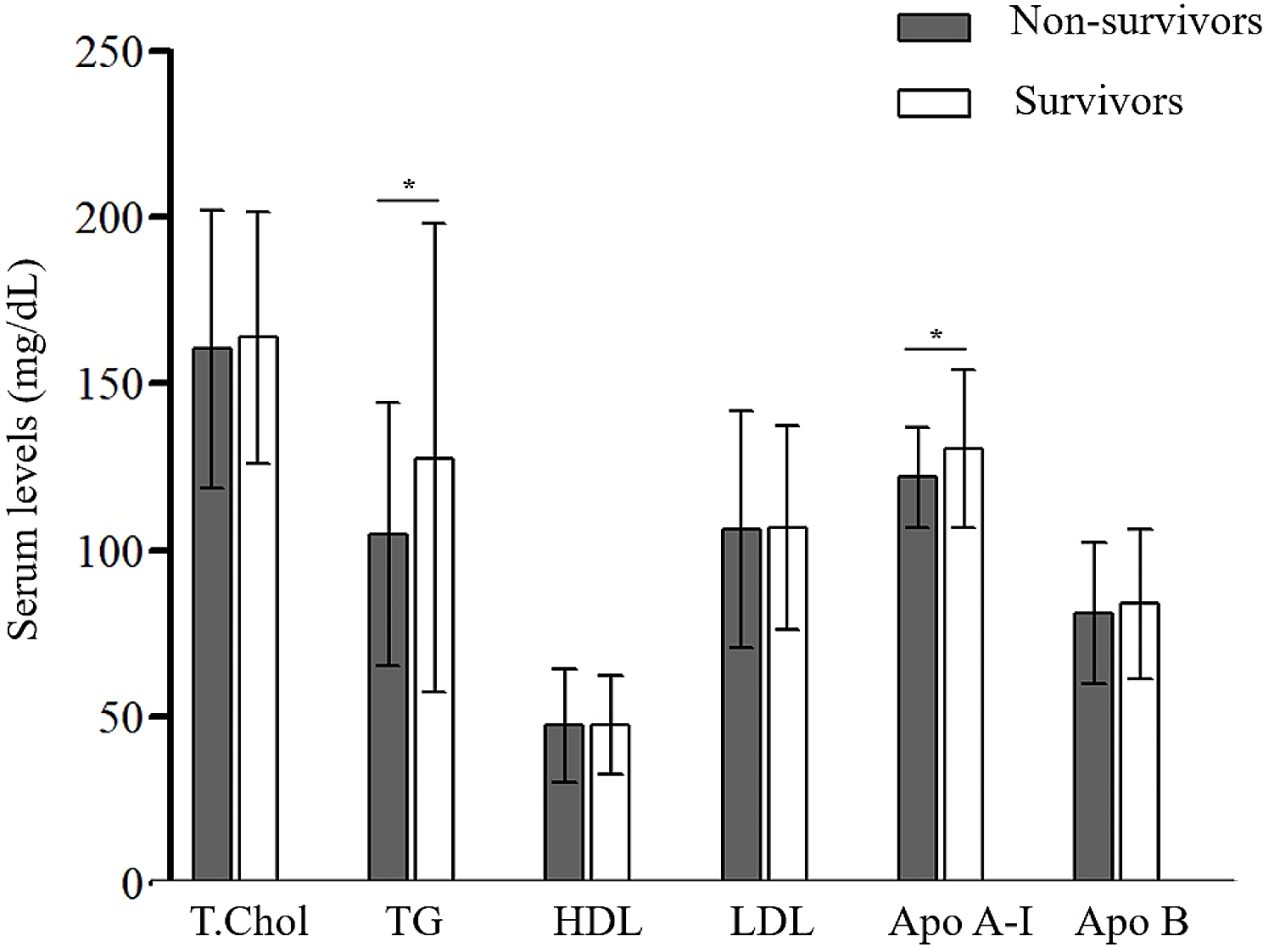
Comparison of serum lipid profiles between the non-survivors and survivors among patients with IPF. *T. Chol* total cholesterol, *TG* triglyceride, *HDL* high-density lipoprotein, *LDL* low-density lipoprotein, *Apo A-I* apolipoprotein A-I, *Apo B* apolipoprotein B. *P < 0.05

In the derivation cohort (*n* = 145), the mean age was 67.5 years and 86.2% were males (Table 1). The median follow-up period was 18.0 months (interquartile range [IQR], 10.0–29.0 months), and 44 (30.3%) patients died. The non-survivors had lower lung function (FVC and DLco) and higher GAP scores than the survivors (Table 1). The mean serum levels of TG (105.0 ± 39.5 vs. 127.7 ± 70.4 mg/dL; *P* = 0.015) and Apo A-I (121.8 ± 15.2 vs. 130.3 ± 23.7 mg/dL; *P* = 0.012) were also lower in the non-survivors than in the survivors (Fig. 2). However, there were no differences in age, proportion of males, smoking history, body mass index (BMI), antifibrotic agent, and statin use between the two groups.

**Table 1:** Baseline characteristics of study patients

Characteristics	Total	Non-survivors	Survivors	*P*-value
Total	145	44	101	
Age, years	67.5 ± 7.6	68.9 ± 8.0	66.9 ± 7.5	0.165
Male	125 (86.2)	36 (81.8)	89 (88.1)	0.432
Ever-smoker	115 (79.3)	31 (70.5)	84 (73.2)	0.117
BMI, kg/m^2^	24.9 ± 3.0	24.5 ± 2.8	25.2 ± 3.1	0.148
FVC (%, predicted)	68.4 ± 15.9	56.5 ± 13.8	73.6 ± 13.9	< 0.001
DLco (%, predicted)	52.8 ± 19.9	39.4 ± 16.3	58.6 ± 18.5	< 0.001
GAP stage				< 0.001
1	66 (45.5)	9 (20.5)	57 (56.4)	
2	59 (40.7)	20 (45.5)	39 (38.6)	
3	20 (13.8)	15 (34.1)	5 (5.0)	
Statin use	45 (31.0)	18 (40.9)	27 (26.7)	0.687
Antifibrotic agent*	111 (76.6)	30 (68.2)	81 (80.2)	0.106

Data are presented as mean ± standard deviation or numbers (%). *BMI* body mass index, *FVC* forced vital capacity, *DLco* diffusing capacity of the lung for carbon monoxide, *GAP* gender-age-physiology

*pirfenidone or nintedanib

### Correlation with clinical parameters

**Fig. 3:**
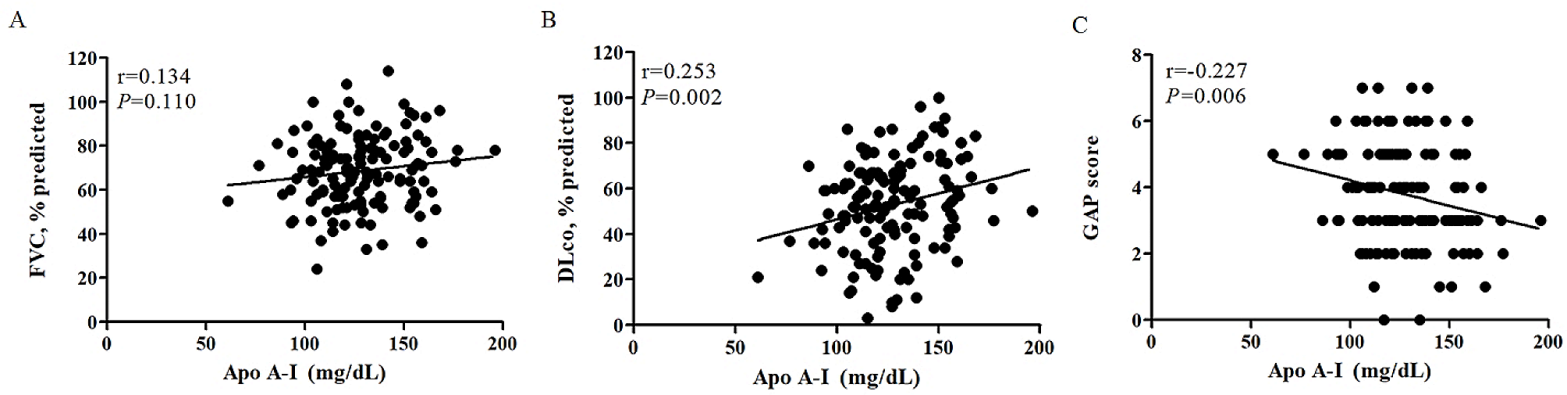
Correlation between apolipoprotein A-I and baseline lung function or GAP score in patients with IPF. Correlation between apolipoprotein A-I and (**A**) FVC, (**B**) DLco, or (**C**) GAP score. *FVC* forced vital capacity, *DLco* diffusing capacity of the lung for carbon monoxide, *GAP* gender-age-physiology, *Apo A-I* apolipoprotein A-I

The serum levels of Apo A-I correlated with DLco (*r* = 0.253; *P =* 0.002) and GAP score (*r* = -0.227; *P =* 0.006) (Fig. 3). LDL levels also showed a significant correlation with GAP score (*r* = -0.181; *P =* 0.029) (see Additional file 1: Table S1). However, total cholesterol, TG, HDL, and Apo B levels were not correlated with FVC, DLco, and GAP scores (see Additional file 1: Table S1).

### Association with the mortality

**Fig. 4:**
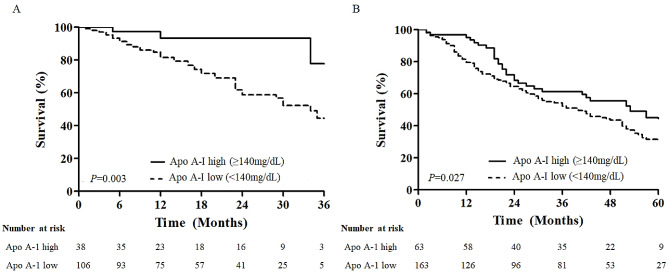
Comparison of Kaplan–Meier between high and low apolipoprotein A-I levels among patients with IPF. (**A**) In the derivation cohort, patients with low Apo A-I levels (< 140 mg/dL, *n* = 107) had higher mortality than those with high Apo A-I levels (≥ 140 mg/dL, *n* = 38) (median survival times, 34.0 ± 3.2 months vs. not reached; *P* = 0.003, log-rank). (**B**) In the validation cohort, patients with low Apo A-I levels (< 140 mg/dL, *n* = 163) had poorer survival than those with high Apo A-I levels (≥ 140 mg/dL, *n* = 63) (median survival times, 40.0 ± 5.6 vs. 53.0 ± 9.5 months; *P* = 0.027, log-rank). *Apo A-I* apolipoprotein A-I

Lipid profiles including total cholesterol, TG, HDL, LDL, Apo A-I, and Apo B levels were converted into categorical variables based on the each cut off values. In the unadjusted Cox regression analysis, never smoker, lower BMI and lung function (FVC and DLco), greater GAP score, low Apo A-I level (< 140 mg/dL) and antifibrotic agent use were significantly associated with mortality in patients with IPF (Table 2). In the multivariate Cox analysis, low Apo A-I level (< 140 mg/dL) was independently associated with mortality (hazard ratio [HR] 3.910, 95% confidence interval [CI] 1.170-13.069; *P* = 0.027), when adjusted by smoking history, BMI, GAP score, and antifibrotic agent use. Patients with a low Apo A-I level (< 140 mg/dL, *n* = 107) showed worse survival (median survival times, 34.0 ± 3.2 months vs. not reached; *P* = 0.003) than those with a high Apo A-I level (≥ 140 mg/dL, *n* = 38) in the Kaplan–Meier survival analysis (Fig. 4A).

**Table 2:** Risk factors for mortality assessed by the Cox proportional analysis in patients with IPF

Characteristics	Unadjusted analysis		Multivariate analysis	
	HR (95% CI)	*P*-value	HR (95% CI)	*P*-value
Age, years	1.029 (0.989–1.070)	0.157		
Male	1.669 (0.737–3.778)	0.219		
Ever-smoker	0.479 (0.245–0.937)	0.032	0.303 (0.152–0.604)	0.001
BMI, kg/m^2^	0.900 (0.813–0.997)	0.043	0.972 (0.864–1.093)	0.633
FVC, predicted %	0.934 (0.914–0.954)	< 0.001		
DLco, predicted %	0.961 (0.948–0.975)	< 0.001		
GAP score	3.934 (2.503–6.184)	< 0.001	2.251 (1.723–2.941)	< 0.001
Statin use	1.192 (0.648–2.193)	0.572		
Antifibrotic agent*	0.422 (0.221–0.804)	0.009	0.240 (0.106–0.545)	0.001
Lipid profiles**				
T. Chol < 200 mg/dL	0.783 (0.385–1.591)	0.499		
TG < 150 mg/dL	1.758 (0.815–3.793)	0.150		
HDL < 40 mg/dL	1.102 (0.600-2.022)	0.755		
LDL < 100 mg/dL	1.141 (0.625–2.084)	0.668		
Apo A-I < 140 mg/dL	4.774 (1.476–15.439)	0.009	3.910 (1.170-13.069)	0.027
Apo B < 80 mg/dL	1.217 (0.667–2.223)	0.522		

*BMI* body mass index, *FVC* forced vital capacity, *DLco* diffusing capacity of the lung for carbon monoxide, *GAP* gender-age-physiology, *T. Chol* total cholesterol, *TG* triglyceride, *HDL* high-density lipoprotein, *LDL* low-density lipoprotein, *Apo A-I* apolipoprotein A-I, *Apo B* apolipoprotein B

*pirfenidone or nintedanib; **converted into dichotomous variables by optimal cutoff values; FVC and DLco were excluded in multivariable Cox analysis because GAP score included FVC and DLco components already

### Validation in the independent cohort

In the validation cohort (*n* = 226), the mean age was 67.2 years and 77.4% were males (see Additional file 1: Table S2). The median follow-up period was 36.0 months (IQR: 15.0–53.0 months) and 138 (61.1%) patients died. The validation cohort showed a lower proportion of males, lower BMI, and less antifibrotic treatment than the derivation cohort; however, Apo A-I levels were not significantly different (128.6 ± 28.4 vs. 127.7 ± 21.7 mg/dL; *P =* 0.744, respectively) between the two cohorts (see Additional file 1: Table S2).

In the validation cohort, the levels of Apo A-I were significantly lower (124.9 ± 29.1 vs. 134.3 ± 26.5 mg/dL; *P* = 0.013) in the non-survivors than in the survivors (see Additional file 2: Figure S1). Apo A-I levels correlated with FVC (*r* = 0.132; *P =* 0.047), DLco (*r* = 0.130, *P =* 0.052), and GAP score (*r* = -0.179; *P =* 0.007) (see Additional file 1: Table S3). Low levels of total cholesterol, TG, LDL, and Apo A-I were significantly associated with mortality in the unadjusted Cox proportional analysis (Table 3). Among them, low Apo A-I level (< 140 mg/dL) was significantly associated with mortality (HR 1.543, 95% CI 1.030–2.313; *P* = 0.036) after adjusting for age and BMI (Table 3). Patients with a low Apo A-I level (< 140 mg/dL, *n* = 163) showed poorer survival (median survival times, 40.0 ± 5.6 vs. 53.0 ± 9.5 months; *P* = 0.027) than those with a high Apo A-I level (≥ 140 mg/dL, *n* = 63) in the Kaplan–Meier survival analysis (Fig. 4B).

**Table 3:** Risk factors for mortality assessed by the multivariate Cox proportional analysis in patients with IPF^†^

Characteristics	Unadjusted analysis		Multivariate analysis*	
	HR (95% CI)	*P*-value	HR (95% CI)	*P*-value
T. Chol < 200 mg/dL	1.681 (1.118–2.528)	0.013	1.316 (0.864–2.003)	0.201
TG < 150 mg/dL	1.619 (1.063–2.467)	0.025	1.299 (0.829–2.034)	0.253
HDL < 40 mg/dL	1.171 (0.829–1.654)	0.370	1.078 (0.755–1.539)	0.679
LDL < 100 mg/dL	1.510 (1.077–2.118)	0.017	1.263 (0.897–1.778)	0.182
Apo A-I < 140 mg/dL	1.562 (1.045–2.336)	0.030	1.543 (1.030–2.313)	0.036
Apo B < 80 mg/dL	1.321 (0.939–1.859)	0.110	1.102 (0.779–1.558)	0.584

*T. Chol* total cholesterol, *TG* triglyceride, *HDL* high-density lipoprotein, *LDL* low-density lipoprotein, *Apo A-I* apolipoprotein A-I, *Apo B* apolipoprotein B

^†^validation cohort; *each variable in multivariable analysis was adjusted by age and BMI

## Discussion

This study suggest that Apo A-I may serve as a valuable blood biomarker for predicting prognosis in IPF; Apo A-I showed a significant correlation with disease severity and was independently associated with mortality in patients with IPF. Patients with low Apo A-I levels also had worse survival than those without. These findings were further validated in an independent IPF cohort.

Our study showed that lower Apo A-I levels were significantly correlated with higher disease severity (lower DLco and higher GAP scores) in patients with IPF. Our results are consistent with previous studies, such as Kim et al. who found lower Apo A-I levels in BAL fluid of 28 patients with IPF compared with 18 healthy controls (median: 7.5 vs. 14.6 ng/mg of BAL protein; *P* < 0.01) and an inverse correlation between lower Apo A-I levels in BAL fluid and higher radiologic fibrosis score (r^2^= -0.23; *P* = 0.031) in IPF [15]. However, our study is the first to report a correlation between blood Apo A-I levels and lung function in IPF, strengthening the potential of Apo A-I levels as a biomarker of disease severity.

Our study demonstrated that Apo A-I levels were independently associated with mortality in patients with IPF and this is consistent with a previous report [22]. In a recent study by Barochia et al., small HDL particle (S-HDLP) was measured using nuclear magnetic resonance spectroscopy in 59 patients with IPF; high level of S-HDLP was associated with a lower incidence of death or lung transplantation in patients with IPF (odds ratio 0.9 for each 1-µmol·L^− 1^ increase in S-HDLP, after adjusting for race, treatment, BMI, and GAP index; *P* < 0.05) [22], which is consistent with the hypothesis that lipids and lipoproteins may influence the prognosis in patients with IPF. In the MESA study investigating subclinical cardiovascular disease in 6,814 normal adults, higher serum levels of Apo A-I were significantly associated with lower HAA on cardiac CT (% change in HAA per standard deviation [SD] of Apo A-I: -1.66, 95% CI -2.36 to -0.97; *P* < 0.001, adjusted for demographics, smoking, and inflammatory biomarkers), and also significantly associated with MMP-7 (% change per SD of Apo A-I [95% CI], -2.83 [-5.32 to -0.27]; *P* = 0.03) and surfactant protein A (% change per SD of Apo A-I [95% CI], -4.79 [-9.56 to 0.12]; *P* = 0.06), which were potential biomarkers of lung inflammation and extracellular matrix remodeling [10, 23, 24]. This finding implies that Apo A-I may play a beneficial role in inhibiting the progression of lung fibrosis and have prognostic implications in patients with IPF.

Furthermore, there is growing interest in understanding the impact of lipids on lung fibrosis. Apo A-I plays an important role in maintaining lipid homeostasis by removing excess cholesterol and phospholipids from various cells, and transporting them to the liver [25]. Additionally, Apo A-I has anti-inflammatory and antioxidant effects [26], which prevent lung injury and fibrosis [12, 15, 16]. Excessive accumulation of lipids such as cholesterol in the cells results in lung inflammation and fibrosis by inducing the expression of collagens and multiple cytokines such as TNF-α, TGF-β1, IL-1, and IL-6 [9]. Moreover, these cytokines could induce the profibrotic signaling in lung parenchyma [13, 14, 27, 28]. Apo A-I facilitates the formation of nascent HDL particles by interacting with ATP-binding cassette transporter A1, enabling the efflux of free cholesterol from the cells. This process helps prevent the overaccumulation of cholesterol in the cells which could lead to lung inflammation and fibrosis [9]. Some studies have suggested the protective effect of Apo A-I on lung inflammation and fibrosis in a murine model [15–17, 29]. In a report by Kim et al., Apo A-I significantly reduced the deposition of bleomycin-induced collagen in a dose-dependent manner when administered to bleomycin-induced mice models [15]. Jiao et al. also demonstrated that Apo A-I decreased the lipoteichoic acid-mediated alveolar damage and acute inflammation in the lung tissues of mice [17].

This study had some limitations. First, this was a single-center retrospective study, which limits the generalizability of our findings. However, it is worth highlighting that the baseline characteristics of our participants resembled those observed in previous studies [15, 22], and the measurements of lipid profile were consistently conducted. Second, there were variations in baseline characteristics such as the proportion of males, BMI, and treatment between the derivation and the validation cohorts. Despite these differences, Apo A-I levels were found to be independently associated with mortality in the multivariable analysis, even after adjusting for potential variables that could influence survival in both cohorts. Third, the adjustment factors in the multivariable Cox analysis differed between the two IPF cohorts, reflecting the differences in baseline characteristics. However, Apo A-I was consistently associated with the mortality in both cohorts after adjustment for the major confounders of IPF survival. Forth, while the study established an association between Apo A-I and all-cause mortality, it did not delve into other important clinical outcomes of IPF, such as acute exacerbation or decline in lung function. Further investigations should explore these endpoints to provide a more comprehensive understanding of the role of Apo A-I in IPF prognosis. Despite these limitations, this study holds substantial value as it is the first study to establish the role of the serum Apo A-I in predicting prognosis in patients with IPF.

In conclusion, our results showed a correlation between serum Apo A-I levels and disease severity in patients with IPF. Furthermore, Apo A-I was identified as independent predictor of mortality in these patients. These results suggest the use of serum Apo A-I as a prognostic biomarker of IPF.

### Electronic supplementary material

Below is the link to the electronic supplementary material.

Supplementary Material 1

Supplementary Material 2

## Data Availability

No datasets were generated or analysed during the current study.

## Abbreviations

Apo A-I: Apolipoprotein A-I
Apo B: Apolipoprotein B
BMI: Body mass index
CT: Computed tomography
DLco: Diffusing capacity of the lung for carbon monoxide
FVC: Forced vital capacity
GAP: Gender-age-physiology
HDL: High-density lipoprotein
IPF: Idiopathic pulmonary fibrosis
ILD: Interstitial lung disease
LDL: Low-density lipoprotein
TG: Triglyceride
