# Supplementary material for: Blood lipid profiles as a prognostic biomarker in idiopathic pulmonary fibrosis
Source: Respir Res. 2024 Jul 18;25:285. doi: 10.1186/s12931-024-02905-z (PMC11264581; doi:10.1186/s12931-024-02905-z)
Supplement: Supplementary file 1 — Supplementary Material 1 [file 12931_2024_2905_MOESM1_ESM.docx]

**Blood lipid profiles as a prognostic biomarker in idiopathic pulmonary fibrosis**

Ju Hyun Oh^1^, Ganghee Chae^2^, Jin Woo Song^3^

**Affiliations:**

^1^Department of Pulmonary and Critical Care Medicine, Sanggye Paik Hospital, Inje University College of Medicine, Seoul, Republic of Korea;

^2^Division of Pulmonary and Critical Care Medicine, Department of Internal Medicine, Ulsan University Hospital, University of Ulsan College of Medicine, Ulsan, Republic of Korea;

^3^Department of Pulmonary and Critical Care Medicine, Asan Medical Center, University of Ulsan College of Medicine, Seoul, Republic of Korea

**Table S1. Correlation of serum lipid profiles and physiologic parameters in patients with IPF**

| **Variables** | | **Correlation coefficient** | ***P*-value** |
| --- | --- | --- | --- |
| Total cholesterol | FVC, % predicted | 0.121 | 0.147 |
|  | DLco, % predicted | 0.049 | 0.560 |
|  | GAP score | -0.152 | 0.069 |
| TG | FVC, % predicted | 0.079 | 0.342 |
|  | DLco, % predicted | -0.047 | 0.574 |
|  | GAP score | -0.039 | 0.643 |
| HDL | FVC, % predicted | 0.006 | 0.938 |
|  | DLco, % predicted | 0.062 | 0.458 |
|  | GAP score | -0.054 | 0.517 |
| LDL | FVC, % predicted | 0.111 | 0.184 |
|  | DLco, % predicted | 0.053 | 0.524 |
|  | GAP score | -0.181 | 0.029 |
| Apo A-I | FVC, % predicted | 0.134 | 0.110 |
|  | DLco, % predicted | 0.253 | 0.002 |
|  | GAP score | -0.227 | 0.006 |
| Apo B | FVC, % predicted | 0.074 | 0.374 |
|  | DLco, % predicted | 0.002 | 0.985 |
|  | GAP score | -0.115 | 0.169 |

FVC, forced vital capacity DLco, diffusing capacity of the lung for carbon monoxide; GAP, gender-age-physiology; TG, triglyceride; HDL, high-density lipoprotein; LDL, low-density lipoprotein; Apo A-Ⅰ, apolipoprotein A-Ⅰ; Apo B, apolipoprotein B;

**Table S2. Comparison of baseline characteristics between the derivation and validation cohorts**

| **Characteristics** | **Derivation cohort** | **Validation cohort** | ***P*-value** |
| --- | --- | --- | --- |
| Total | 145 | 226 |  |
| Age, years | 67.5 ± 7.6 | 67.2 ± 7.8 | 0.669 |
| Male | 125 (86.2) | 175 (77.4) | 0.042 |
| Ever-smoker | 115 (79.3) | 165 (73.0) | 0.176 |
| BMI, kg/m^2^ | 24.9 ± 3.0 | 24.0 ± 3.1 | 0.003 |
| FVC (%, predicted) | 68.4 ± 15.9 | 67.5 ± 17.6 | 0.615 |
| DLco (%, predicted) | 52.8 ± 19.9 | 54.9 ±19.0 | 0.291 |
| GAP index |  |  | 0.504 |
| 1 | 66 (45.5) | 110 (48.7) |  |
| 2 | 59 (40.7) | 79 (35.0) |  |
| 3 | 20 (13.8) | 37 (16.4) |  |
| Statin use | 45 (41.7) | 107 (47.3) | 0.349 |
| Antifibrotic agent* | 111 (76.6) | 125 (55.3) | 0.001 |
| Lipid profiles |  |  |  |
| Total cholesterol | 162.8 ± 38.9 | 174.7 ± 38.4 | 0.004 |
| Triglyceride | 120.8 ± 63.4 | 125.6 ± 85.1 | 0.543 |
| HDL | 47.3 ±15.4 | 47.5 ± 16.7 | 0.916 |
| LDL | 106.5 ± 32.2 | 109.2 ± 33.1 | 0.434 |
| Apolipoprotein A-I | 127.7 ± 21.7 | 128.6 ± 28.4 | 0.744 |
| Apolipoprotein B | 83.0 ± 21.9 | 90.0 ± 21.9 | 0.003 |

Data are presented as mean ± standard deviation or numbers (%). BMI, body mass index; FVC, forced vital capacity; DLco, diffusing capacity of the lung for carbon monoxide; GAP, gender-age-physiology; HDL, high-density lipoprotein; LDL, low-density lipoprotein; *pirfenidone or nintedanib

**Table S3. Correlation of serum lipid profiles and physiologic parameters in patients with IPF^†^**

| **Variables** | | **Correlation coefficient** | | ***P*-value** |
| --- | --- | --- | --- | --- |
| Total cholesterol | FVC, % predicted | | 0.108 | 0.105 |
|  | DLco, % predicted | | 0.151 | 0.024 |
|  | GAP score | | -0.238 | <0.001 |
| TG | FVC, % predicted | | 0.051 | 0.449 |
|  | DLco, % predicted | | 0.043 | 0.517 |
|  | GAP score | | -0.084 | 0.209 |
| HDL | FVC, % predicted | | 0.023 | 0.727 |
|  | DLco, % predicted | | 0.058 | 0.384 |
|  | GAP score | | -0.100 | 0.136 |
| LDL | FVC, % predicted | | 0.057 | 0.396 |
|  | DLco, % predicted | | 0.096 | 0.150 |
|  | GAP score | | -0.159 | 0.016 |
| Apo A-I | FVC, % predicted | | 0.132 | 0.047 |
|  | DLco, % predicted | | 0.130 | 0.052 |
|  | GAP score | | -0.179 | 0.007 |
| Apo B | FVC, % predicted | | 0.073 | 0.272 |
|  | DLco, % predicted | | 0.064 | 0.339 |
|  | GAP score | | -0.143 | 0.033 |

FVC, forced vital capacity DLco, diffusing capacity of the lung for carbon monoxide; GAP, gender-age-physiology; TG, triglyceride; HDL, high-density lipoprotein; LDL, low-density lipoprotein; Apo A-Ⅰ, apolipoprotein A-Ⅰ; Apo B, apolipoprotein B; ^†^validation cohort
