# Supplementary material for: Blood lipid profiles as a prognostic biomarker in idiopathic pulmonary fibrosis
Source: Respir Res. 2024 Jul 18;25:285. doi: 10.1186/s12931-024-02905-z (PMC11264581; doi:10.1186/s12931-024-02905-z)

**Blood lipid profiles as a prognostic biomarker in idiopathic pulmonary fibrosis**

Ju Hyun Oh^1^, Ganghee Chae^2^, Jin Woo Song^3^

**Affiliations:**

^1^Department of Pulmonary and Critical Care Medicine, Sanggye Paik Hospital, Inje University College of Medicine, Seoul, Republic of Korea;

^2^Division of Pulmonary and Critical Care Medicine, Department of Internal Medicine, Ulsan University Hospital, University of Ulsan College of Medicine, Ulsan, Republic of Korea;

^3^Department of Pulmonary and Critical Care Medicine, Asan Medical Center, University of Ulsan College of Medicine, Seoul, Republic of Korea

**Figure S1. Comparison of serum lipid profiles between the non-survivors and survivors among patients with IPF^†^**

T. Chol, total cholesterol; TG, triglyceride; HDL, high-density lipoprotein; LDL, low-density lipoprotein; Apo A-I, apolipoprotein A-I; Apo B, apolipoprotein B; * *P* <0.05; ^†^validation cohort


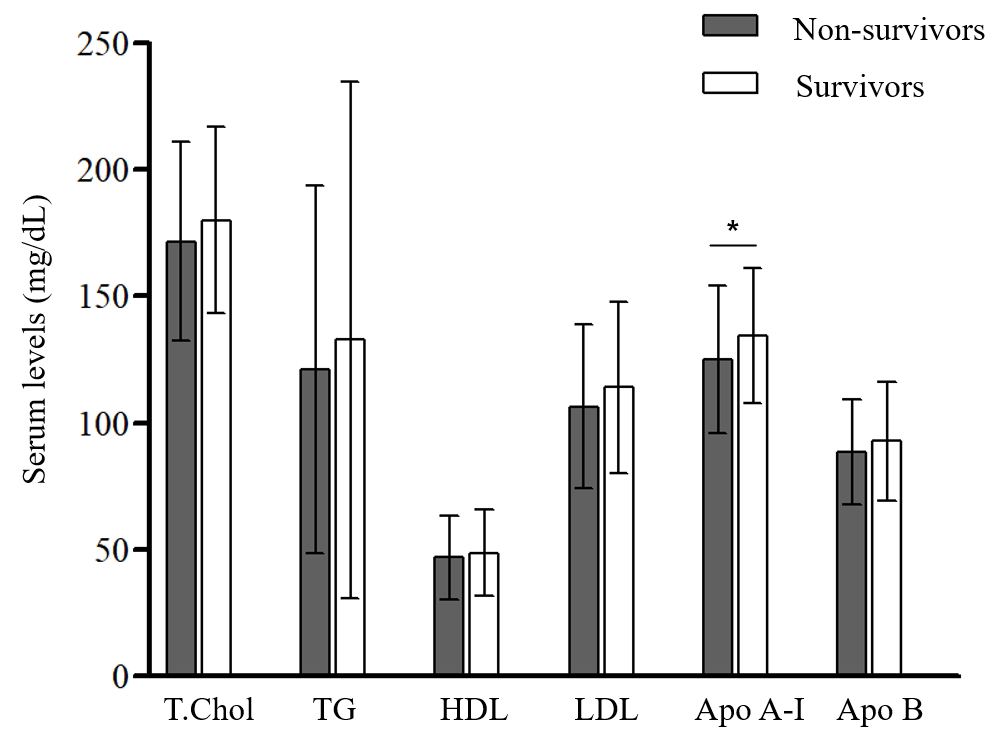

Supplement: Supplementary file 2 — Supplementary Material 2 [file 12931_2024_2905_MOESM2_ESM.docx]
